# Pancake kidney: when it is not a problem

**DOI:** 10.1259/bjrcr.20170117

**Published:** 2018-02-16

**Authors:** Milena Pasquali, Nicola Sciascia, Giovanni D'Arcangelo Liviano, Gaetano La Manna, Maurizio Zompatori

**Affiliations:** 1 Department of Experimental, Diagnostic and Specialty Medicine (DIMES), Division of Radiology, Policlinico Sant Orsola-Malpighi, Bologna, Italy; 2 Department of Radiology Cardiothoracic section, Policlinico Sant Orsola-Malpighi, Bologna, Italy; 3 Department of Experimental, Diagnostic and Specialty Medicine, Nephrology, Dialysis and Transplantation Unit, Policlinico Sant Orsola-Malpighi, University of Bologna, Bologna, Italy; 4 Department of Radiology, San Giuseppe Hospital - MultMedica IRCCS, Milano, Italy

## Abstract

Pancake kidney is a very rare congenital anomaly involving complete fusion of medial renal parenchyma. The interface is devoid of any intervening septum. As described, the kidneys form a single lobulated mass in pelvic location. However, dual collecting systems are retained, and the shortened, anteriorly seated ureters enter the bladder normally. This condition is usually discovered incidentally but may confer a heightened susceptibility to recurrent urinary tract infections or stone formation, given the likelihood of anomalous collecting system rotation and the potential for ureteral stasis or obstruction. Excretory urography, the customary method of detection, has been replaced by ultrasonography, CT, MRI, and radionucleotide scanning. Herein, we present a male patient with a pelvic pancake kidney, never symptomatic. A conservative approach of regular follow up visits and laboratory testing was elected, thus avoiding any unnecessary investigations or extensive surgery.

## Clinical Presentation

A 47-year-old male presented to our department for CT urography. This patient denied any history of kidney disease, whether in childhood or as an adult. However, in the course of appendectomy at 8 years of age, a renal anomaly encountered was arbitrarily classified as horseshoe kidney by the attending surgeon. No further diagnostic studies were pursued until September 2015, at which time his primary care physician was consulted to certify competitive sports eligibility. He was then seen in referral by a nephrologist who initiated a battery of diagnostic tests.

## Clinical Investigation/Imaging Findings

**Figure 1.**
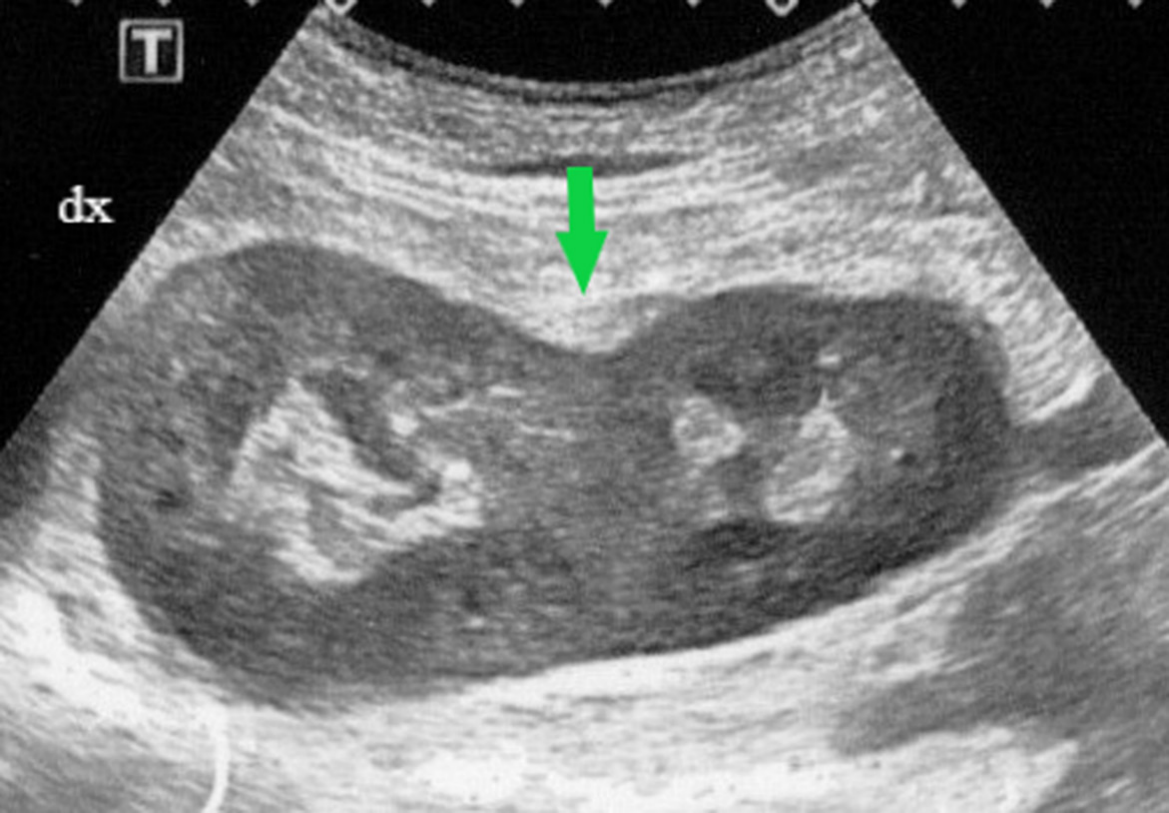
Ultrasound image (axial view): fusion of kidneys at medial parenchymal margins (green arrow).

**Figure 2.**
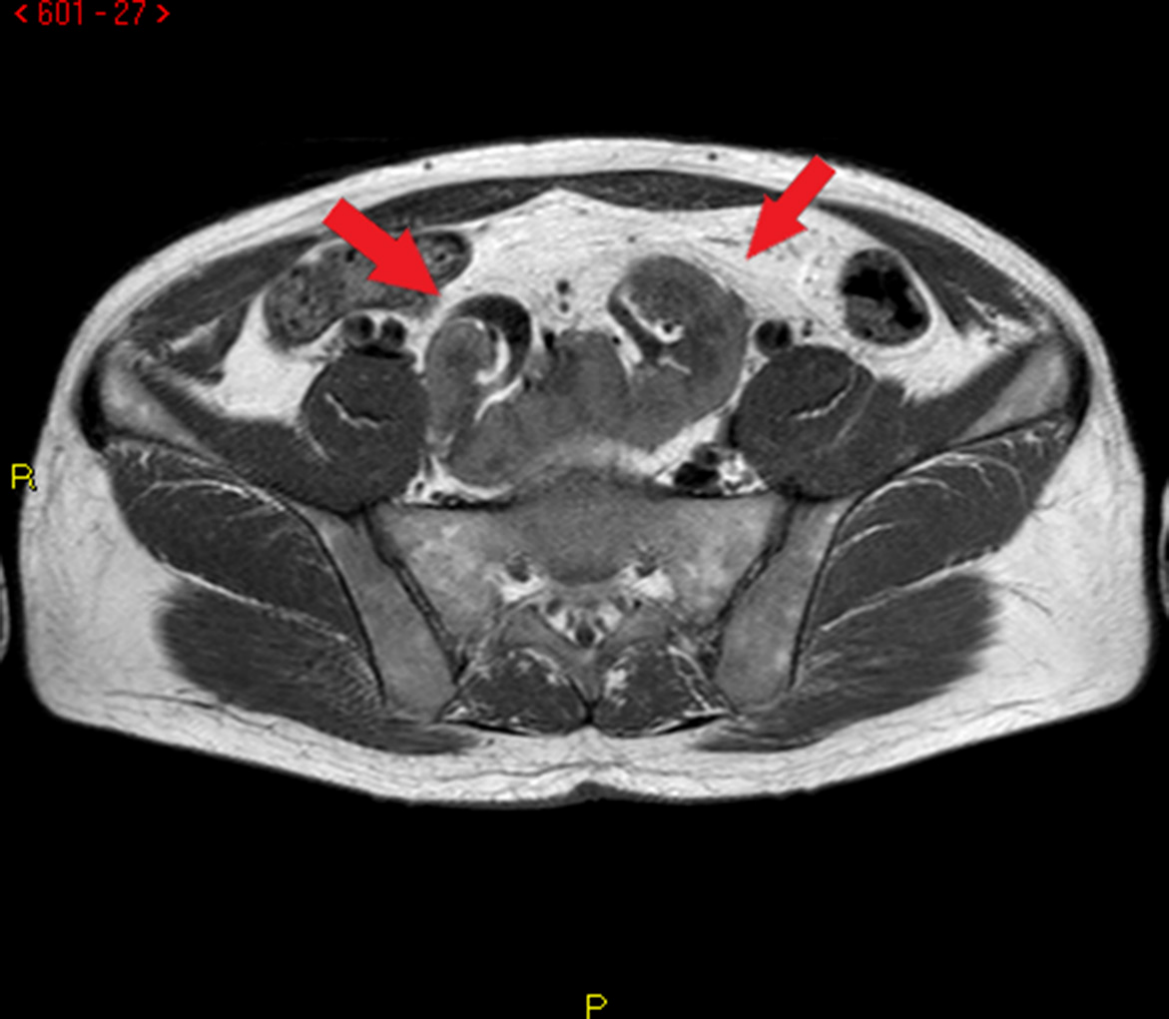
MRI (TSE *T*_1_ weighted): pelvic location of pancake kidney in axial view (arrows). TSE, turbo spin echo.

**Figure 3.**
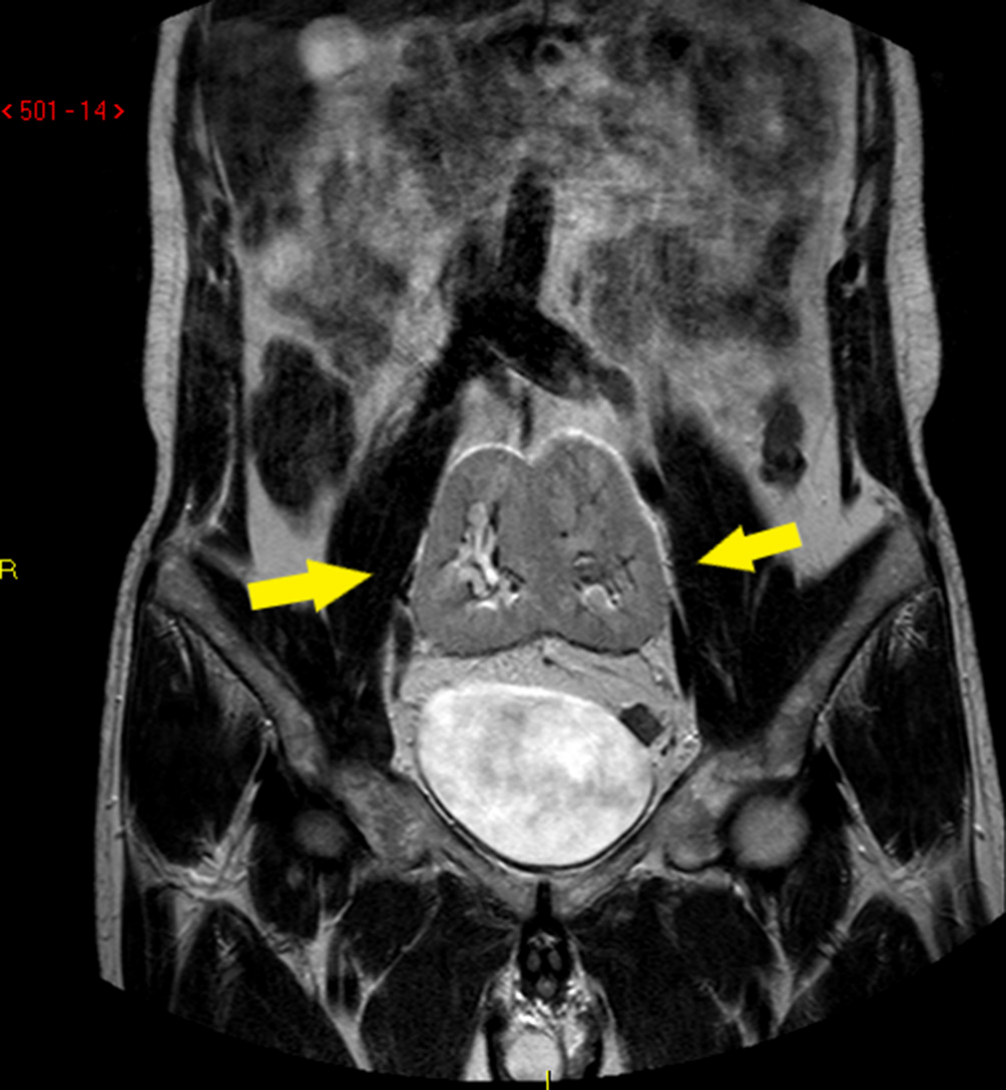
MRI (TSE *T*_2_-weighted): pelvic location of pancake kidney in coronal view (arrows). TSE, turbo spin echo.

The nephrologist had requested an array of laboratory tests to assess kidney function, as well as abdominal ultrasound and MRI. All laboratory testing proved unremarkable, and the patient’s blood pressure was normal. Ultrasound (Figure 1) and MRI (Figures 2 and 3) studies indicated that the kidneys were fused medially and sat within the pelvic cavity, constituting a case of pancake kidney. Upon request of the nephrologist, CT urography was also undertaken at our facility to better define the renovascular origins and urinary tract anatomy.

**Figure 4.**
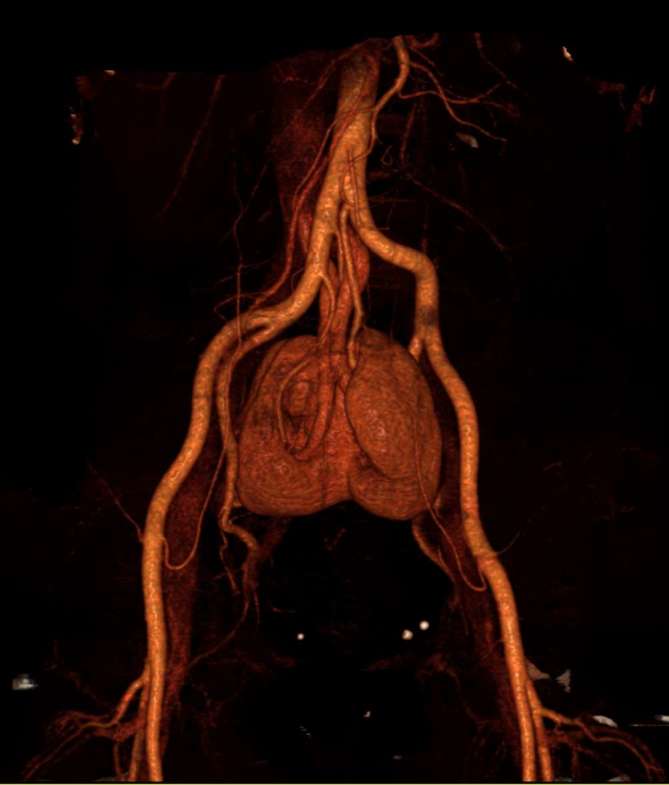
CT 3D volume rendering of vascular supply to pancake kidney: dual arteries (each side), originating from ipsilateral common iliac artery; and dual veins (each side), leading to confluence of iliac veins. 3D, three-dimensional.

**Figure 5.**
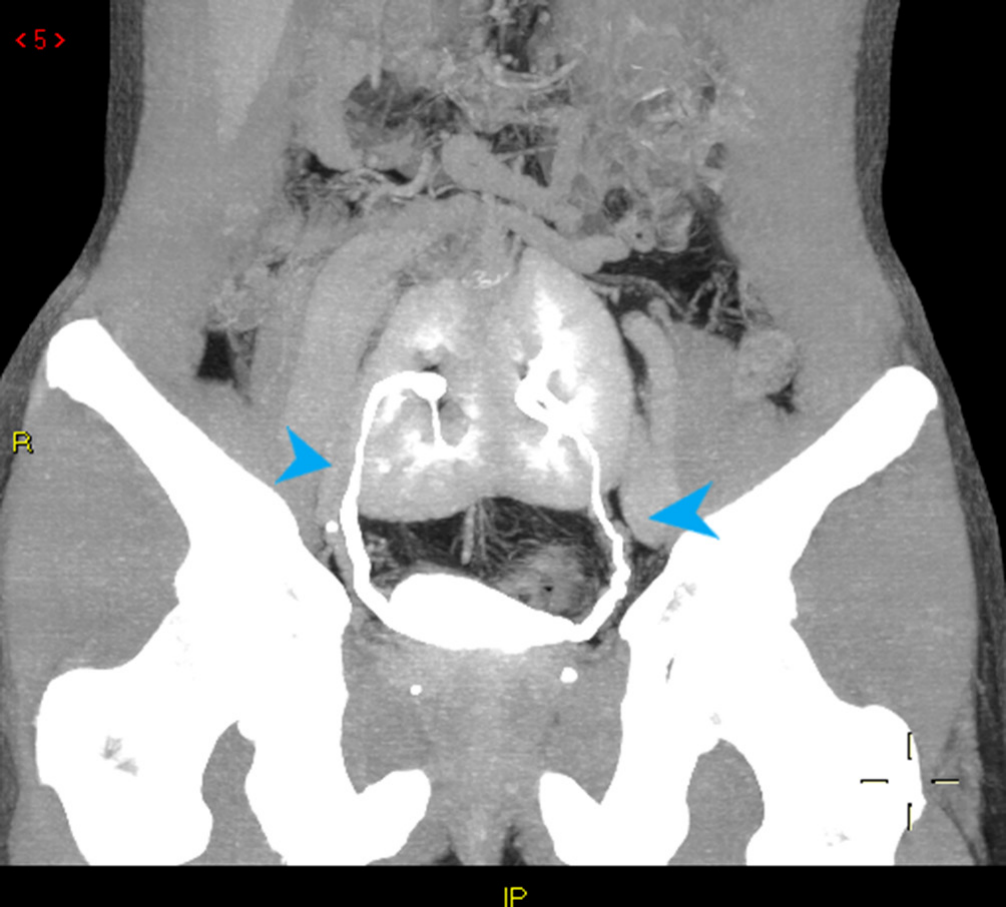
CT MIP coronal reconstruction of urinary tract anatomy: two ureters entering normally into bladder (tip of arrows). MIP, maximum intensity projection.

Ultimately, pancake kidney was confirmed, marked by a medially fused renal mass within the pelvis, near sacral promontory. Separate dual excretory systems were also verified anterior to the renal mass. Otherwise, parenchymal and corticomedullary development were not unusual. Post-processing CT images fully depicted both vascular supply and urinary tract anatomy.(Figures 4–5)

## Treatment and Outcome

Currently, this patient remains asymptomatic. Although he is subject to long-term follow-up surveillance, no treatment is presently required, and he is expected to lead a normal life.

## Discussion

Congenital anomalies of the kidney and urinary tract occur in 3.3–11.1% of the population, accounting for ~50% of all congenital defects. Collectively, renal malformations are clinically challenging disorders in which imaging is pivotal for early diagnosis and proper management.^1^ Within the realm of renal fusion anomalies, there are two principal types: horseshoe kidney and pancake kidney.

Horseshoe kidney is one of the most frequent renal malformations, with an incidence of 0.25% in the general population. Typically, the lower poles of the kidneys are fused and are more medially aligned than the upper poles, seated at L3-5 (lumbar) vertebral level anterior to the great vessels. The renal isthmus may be parenchymal or fibrous in nature, approximating the junction of aorta and inferior mesenteric artery.^2^

By comparison, pancake kidney is an extremely rare congenital fusion anomaly, described as a lobulated renal mass displaced to pelvis. There is complete medial parenchymal fusion, with no intervening septum. Each kidney has its own collecting system, and the shortened, anteriorly located ureters enter the bladder normally.^3^ This anomaly is also referred to as cake, disc, doughnut, or shield kidney, because it results in a ring- or doughnut-shaped mass.^4^

In the published literature, there are no precise figures for the incidence or prevalence of pancake kidney. Miclaus and colleagues have estimated that 1 in 65,000–375,000 individuals are affected.^5^ Numerous embryologic errors, including faulty ureteral bud development, aberrant renovascular phenotypes (that limit kidney ascent), and teratogenic factors, have been implicated in the very early gestational events leading to positional and fusion anomalies.^6^ Cook and Stephens have argued that anomalies of this sort reflect abnormal variants of hind-end growth or flexion in the developing embryo.^7^

Another theory is based on the mechanics of renal fusion, namely a pressing together of nephrogenic primordia by the umbilical arteries during ascent from the pelvis.^8^ The human kidneys arise from metanephric blastema within the pelvis, later transitioning to lumbar position. They will also undergo lateral shift and deflection and internal rotation in the course of migration. Prior to this mobilization, however, a critical push between the two umbilical arteries is initiated. It is at this point where fusion is apt to occur, creating a single renal mass that fails to properly ascend.^9^

Despite all conjecture, the fundamentals of renal fusion anomalies have yet to be explained with certainty. It is clear, however, that in our patient the kidneys were never positioned any higher, given the short ureters and locally configured arterial blood supply. Furthermore, pancake kidney appears more common in males (M:F ratio, 2–3:1); and although patients may present at any age, most are between 30–60 years old at diagnosis.^8^ This anomaly may also accompany other congenital defects, such as testicular descent or vas deferens anomalies, vaginal agenesis, bi- or unicornuate uterus, sacral agenesis, caudal regression syndrome, tetralogy of Fallot, and spina bifida.

The vascular supply in such patients may arise from one or more renal arteries (distal aortic or iliac branches) and one or more renal veins (tributaries of inferior vena cava or iliac vein).^9^ If only single vessels are involved, the risk of compromise by pelvic trauma, pregnancy, or space occupying lesions is increased. Moreover, atherosclerosis of the aorta and iliac arteries is a hazard in old age, causing stenosis of aberrant renal arteries and possibly hypertension. It is, therefore essential to monitor blood pressure when managing these patients.^10^

Discovery of pancake kidney is usually incidental. Excretory urography, the customary method of detection, has been replaced by ultrasonography, CT or MR urography, and radionucleotide scanning. Ultrasonography is a non-invasive modality of greater benefit in pre- or post-natal appraisal of renal anomalies, whereas multidetector CT (MDCT) urography is especially suited for depicting urinary tract anatomy, including renal parenchyma, collecting system, and ureters.^11^ Post-processing images of multidetector CT using maximum intensity projection, volume rendering, and multiplanar reconstruction techniques provide a sense of three-dimensionality and result in greater diagnostic . In our CT studies, we have applied all of these techniques to facilitate the delineation of anatomic anomalies and help specialty physicians (*i.e.* nephrologists, urologists) better appreciate various structural and vascular configurations.

Patients with pancake kidney are prone to recurrent urinary tract infections and stone formation, given the likelihood of anomalous collecting system rotation and the potential for ureteral stasis or obstruction. Consequently, they may present with signs of urinary tract infection, fever, and vague lower abdominal pain.^4^ However, most are asymptomatic, as was our patient. Although he denied any family history of kidney disease or any childhood bouts of kidney disease, we did find a small triangular-shaped cortical scar by CT, attesting to prior pyelonephritis. The pancake kidney is also susceptible to injury during pelvic surgery, particularly aortic procedures. Because the blood supply originates from aortic bifurcation or iliac vessels, the entire kidney is rendered ischemic during proximal aortic cross-clamping.^10^

The finding of pancake kidney does not readily doom a patient to progressive renal failure. In the event that symptoms of renal failure develop, renal function tests turn abnormal, urinary outflow obstruction is evident, or obstructive uropathy ensues, surgery is perhaps warranted. However, parenchymal separation carries a risk of renovascular damage, kidney necrosis or infarction, or post-operative renal failure.^3^ In addition, there is evidence that the relative risk of developing various primary neoplasms, including Wilms tumour, renal cell carcinoma, or rarely rhabdomyosarcoma, is heightened in patients with renal fusion anomalies.^12^

## Conclusion

In an asymptomatic patient such as this, pancake kidney can be managed conservatively through long-term monitoring of renal function, remaining vigilant for infectious and obstructive complications or stone formation. Unnecessary investigations and extensive surgery are thereby avoided, and the patient may anticipate a normal lifestyle. The follow-up plan is best established by a nephrologist, based on clinical and laboratory findings.

In conclusion, pancake kidney is not uniformly problematic, despite the seeming gravity of this rare congenital anomaly.

## Learning points

1. Pancake kidney is rare congenital fusion anomaly of the kidney.
2. Discovery is usually incidental. MDTC urography and MR urography help accurately define the urinary tract anatomy, including renal parenchyma, collecting system, ureteral configuration, and vascular supply.
3. Asymptomatic patients can be managed conservatively (*i.e.* without specific treatment) through long-term follow-up monitoring of renal function.
